# Fisurectomy and anoplasty with botulinum toxin injection in patients with chronic anal posterior fissure with hypertonia: a long-term evaluation

**DOI:** 10.1007/s13304-020-00846-y

**Published:** 2020-07-14

**Authors:** Beatrice D’Orazio, Girolamo Geraci, Guido Martorana, Carmelo Sciumé, Giovanni Corbo, Gaetano Di Vita

**Affiliations:** 1 General Surgery Unit, Department of Surgical, Oncological and Stomatological Sciences, University of Palermo, Via Liborio Giuffrè, 5, 90127 Palermo, Italy; 2 Postgraduate Medical School in General Surgery, University of Palermo, Palermo, Italy; 3 General and Oncological Surgery Unit, Fondazione Istituto G. Giglio, Cefalù, Italy

**Keywords:** Proctology, Anal fissure, Fissurectomy, Anoplasty, Sphincterotomy, Botulinum toxin

## Abstract

Chronic anal fissure’s (CAF) etiopathogenesis remain unclear. CAF of the posterior commissure (CAPF) are often characterized by internal anal sphincter (IAS) hypertonia. The treatment of this disease aimed to reduce IAS hypertonia. Due to the high rate of anal incontinence after LIS, the employment of sphincter preserving surgical techniques associated to pharmacological sphincterotomy appears more sensible. The aim of our study is to evaluate the long-term results of fissurectomy and anoplasty with V–Y cutaneous flap advancement associated to 30 UI of botulinum toxin injection for CAPF with IAS hypertonia. We enrolled 45 patients undergone to fissurectomy and anoplasty with V–Y cutaneous flap advancement and 30 UI botulinum toxin injection. All patients were followed up for at least 5 years after the surgical procedure, with evaluation of anal continence, recurrence rate and MRP (Maximum resting pressure), MSP (Maximum restricting pressure), USWA (Ultrasound wave activity). All patients healed within 40 days after surgery. We observed 3 “de novo” post-operative anal incontinence cases, temporary and minor; the pre-operative ones have only temporary worsened after surgery. We reported 3 cases of recurrences, within 2 years from surgery, all healed after conservative medical therapy. At 5 year follow-up post-operative manometric findings were similar to those of healthy subjects. At 5 years after the surgical procedure, we achieved good results, and these evidences show that surgical section of the IAS is not at all necessary for the healing process of the CAPF.

## Introduction

Chronic anal fissure (CAF) is one of the most frequent proctological disease, its clinical features are post-defecation bleeding, itching and pain, which can last from few minutes–hours. CAF most frequently occur at the posterior commissure (CAPF) [1], nevertheless, to present the etiopathogenesis of the disease remains unclear. CAPF are characterized, more often than CAF at the anterior commissure, by internal anal sphincter (IAS) hypertonia. To date, the role of this latter feature is unknown, and we still wonder whether it is a cause or a consequence of CAF disease. During the last few decades, the treatment of this disease aimed to reduce IAS hypertonia with medical or surgical techniques. Surgical lateral internal sphincterotomy (LIS) represents nowadays the gold standard treatment for CAFs, which are refractory to the pharmacological therapy; this surgical procedure is burdened by low rate of post-operative complications and high healing rate. The great disadvantage of the latter procedure is the high rate of anal incontinence, which can occur in up to 30–40% of cases. A recent meta-analysis [2] evaluating long-term incidence of anal incontinence after LIS, showed an overall continence alteration risk of 14%; however, on severity analysis, flatus incontinence and soilage/seepage were much commoner than frank incontinence to liquid or solid stool.

Anal incontinence has a strong impact on the quality of life of patients and it can be more disabling than CAF itself [3]; as a matter of fact, patients tend to bear better the recurrence than the fecal incontinence [4].

To preserve the anatomical and functional integrity of the sphincterial system as well as to reduce the anal incontinence incidence, the surgical procedures mostly used for the treatment of CAPF are fissurectomy alone or fissurectomy and pharmacological sphincterotomy, which can be associated with cutaneous and mucous flap.

The aim of our study is to evaluate the results of fissurectomy and anoplasty with V–Y cutaneous flap advancement associated to 30 UI of botulinum toxin injection, at 5 years after the sphincter-saving surgical procedure, as a treatment for CAPF with IAS hypertonia.

## Materials and methods

We enrolled 45 patients, all affected by idiopathic and non-recurrent CAPF with hypertonic IAS, who underwent fissurectomy and anoplasty with V–Y cutaneous flap advancement and 30 UI botulinum toxin injection, from January 2011 until January 2015. All patients were followed up for at least 5 years after the surgical procedure. The patients’ outcome data were retrieved from a prospectively monitored database.

Pre-operative manometric evaluation was performed after a reasonable period of suspension of all medical therapy influencing IAS tone. The manometric evaluation was carried out by a manometric sensor (2.1 mm external diameter) with four circle orifices and a latex microbaloon at its extremity (Marquat C87; Boissy, St-Leger, France). The machine was connected to a polygraph (Narco; Byosystem MMS 200, Houston TX) using the station pull-through method with perfusion of normal saline and the patient lying on the right side. At manometric evaluation, maximum resting pressure (MRP) and maximum squeeze pressure (MSP) were defined as the maximum pressure detected, respectively, on resting and after voluntary contraction. Ultraslow wave activity (USWA) was defined as pressure’s waves with frequency of less than 2/min and an amplitude greater than 25 cm H_2_O [5].

Data collected on healthy subjects by our anorectal pathophysiological laboratory showed [6] that the normal values of MRP and MSP were, respectively, 68.1 ± 12.3 mmHg mmHg and 112 ± 36,2 mmHg; USWA was detected in the 10% of patients. In accordance to Jones et al. [7], normal range of MRP was 45–85 mmHg; so that CAAF with hypertonic IAS were defined as those with MRP values > 85 mmHg. Manometric follow-up was performed at 12 and 60 months after the surgery.

All patients underwent fissurectomy and anoplasty with V–Y skin flap advancement lying in a gynecological position under spinal or general anesthesia. To expose the anal canal we used four Kocher pliers placed at 3, 6, 9 an 12 h to avoid employing anal retractors; an Eisenhammer retractor or a speculum have been gently introduced just in case of necessity.

After injection of 5 ml of local anesthetic solution (100 mg hydrochloride mepivacaine an 0.025 mf l-adrenaline), the fibrotic edges were excised with a scalpel until normal non-fibrotic anodermal tissue showed sufficient bleeding. The sentinel skin tag and hypertrophied papilla at the level of dentate line were excised when present according to Gupta and Kalaskar [8]. The tissue at the base of the fissure was curetted until there were clean muscle fibers of the IAS. There was no use of diathermy and careful attention was payed to avoiding damages of the IAS. Standard advancement anoplasty was performed using a flap of healthy skin tissue which was mobilized and then advanced with its blood supply to fill in the defect. The flap was secured without tension to the anal canal and the skin was closed tension free in a V–Y manner with interrupted rapid absorbable suture behind the advancement flap. Once the fissurectomy and anoplasty was performed, the botulinum toxin (BT) A (Botox, Allergan, Westport, Ireland) store at − 20 °C and diluted in saline to 50 UI/ml was injected into IAS with a 27-gauge needle. Each patient received a total of 30 UI of BT: 15 UI injected at 3 h in gynecological position and 15 UI injected at 9 h. None of the patients assumed concomitant oral medications that could interfere with the action of type A BT (aminoglycosides, baclofen, dantrolene, diazepam) and there was no known hypersensitivity to any component of BT formulation. All procedures were carried out by the same senior surgeon (GDV). Before surgery, all patients received a small volume of phosphate–saline enema. Metronidazole was administered intravenously in a dose of 500 mg 1 h before surgery. Subsequently, it was administered per os at the dosage of 250 mg for 7 days, three times daily.

During the first 2 weeks after the surgery, patients took variable doses of psyllium fibers. A laxative preparation (sennosides) was given orally to subjects who had not yet passed stools 3 days after surgery. Enema, suppositories and all rectal manipulation were avoided. Immediately after surgery, all patients received 100 mg of diclofenac intramuscularly for analgesia and were instructed to take only 100 mg of nimesulide tablets when needed. The primary goal of the study was the patient’s complete healing and the evaluation of incontinence and recurrence rate; the secondary goal included the evaluation of MRP, MSP, USWA, symptom relief (bleeding, itching and pain) and recording pro forma of the immediate an long time complications related to flap (anal stenosis, keyhole deformity, urinary retention, related side effects of BT). A complete healing was defined as a complete epithelialization of the advancement skin flap. Both duration and intensity of pain post-defecation were evaluated. Pain intensity was scored with a visual analogical scale (VAS) from 0 to 10, where 0 corresponded to no pain and 10 to the worst pain conceivable. Anal incontinence was assessed pre-operatively and after 1, 3, 6, 12 and 60 months from surgery using the Pescatori grading system [9]: A incontinence for flatus and mucus; B for liquid stool; C for solid stool; 1 for occasional; 2 for weekly and 3 for daily. Patients were discharged 24 h after surgery, afterwards they were examined until they were completely healed and they were also followed up at 1, 12 and 60 months following the surgical procedure. Independently of the scheduled appointments, patients were seen on request.

### Statistical analysis

Continuous variables were expressed as a mean with standard deviation and qualitative data as absolute frequencies, MRP values were also given as median and range. Student’s *t* test with Welch correction was used to analyze the differences of pain score and pain duration at each registration point. Values of *P* < 0.05 were considered statistically significant.

## Results

This study includes 15 women and 30 men. At the time of surgical procedure, the median age of the patient was 39 years (range 18–72). Bowel function was normal in 13 patients, 26 patients suffered from constipation and 6 from diarrhea; bowel function was assessed according to the updated Rome diagnostic criteria. Seven women were nulliparous, 4 gave natural birth and all of them underwent an episiotomy and 4 patients gave birth throughout a caesarean section. Clinical features of AF are reported in Table 1.

**Table 1:** Characteristics of Chronic Anal fissure

	No.	Per cent
Hypertrophied anal papilla	36	80
Skin tags	30	66,6
Symptoms		
Pain	45	100
Bleeding	34	75,5
Pruritus	22	48,8
Duration of Symptoms Months (mean ± standard deviation)	20,1 ± 15,2	

### Healing fissure and relief of symptoms

**Fig. 1:**
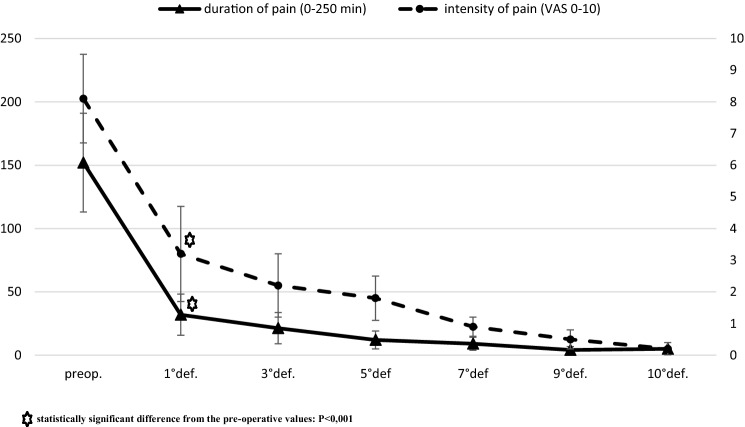
Intensity and duration of pain related to defecation after fissurectomy and anal advancement. Star statistically significant difference from the pre-operative values: *P* < 0.001

We achieved complete wound healing, in all patients, within 40 days after surgery. Intensity and duration of post-defecation pain was significantly reduced with respect to the pre-operative values starting from the first defecation (*P* < 0.001) (Fig. 1). None of the patients complained about pain, bleeding or itching 40 days after surgery. Analgesics consumption decreased significantly after first defecation.

### Incontinence

We recorded 3 cases of pre-operatory anal incontinence (6.6%), 2 cases were type A1 and one was type A2 according to Pescatori grading system. In 2 of these patients, anal incontinence resulted worsened at 1 month follow-up after surgery, while at 3 month follow-up, the grading score was similar than the pre-operative recorded one, Table 2. We observed only 3 “de novo” anal incontinence cases but in all of them the alteration was minor and temporary, Table 3.

**Table 2:** Evaluation of pre-operative anal incontinence cases according to Pescatori grading system from 1 month to 60 months after the surgical procedure

Pre-operative Anal incontinence cases	1 month after the procedure	3 months after the procedure	6 months after the procedure	12 months after the procedure	60 months after the procedure
A2	A2	A2	A2	A2	A1
A1	A2	A1	A1	A1	A1
A1	A2	A1	–	–	A1

**Table 3:** Evaluation of post-operative “de novo” anal incontinence cases according to Pescatori grading system from 1 to 60 months after the surgical procedure

Post-operative “de novo” Anal incontinence cases	1 month after the procedure	3 months after the procedure	6 months after the procedure	12 months after the procedure	60 months after the procedure
1	A1	–	–	–	–
**2**	A1	A1	–	–	–
**3**	A2	A1	–	–	–

### Recurrences

We reported only 3 cases of recurrences, 2 women and 1 man, the site of the recurrences was always different from the primary location. All of them occurred within 2 years from surgery and all were characterized by the persistence of IAS hypertonia. All patients underwent a medical treatment consisting in implementation of fibers in the diet, employ of local products containing nifedipine or lidocaine and anal dilators. All patients responded to the conservative treatment with a complete healing.

### Manometry findings

Pre-operative values of MRP were significantly higher as compared with healthy subjects (101 ± 15 mmHg; *P* = 0.001), whereas MSP values were only slightly increased (125 ± 16.8 mmHg; *P* = n.s.).

At 12 months and 60 months after surgery MSP values did not significantly differ as compared both with pre-operative levels and healthy control subjects.

At 12 month follow-up, MRP values were significantly lower as compared with pre-operative values (*P* < 0.001). As compared with healthy controls, MRP values had still increased (*P* < 0.01). At 60 months, MRP values were similar compared with healthy control subjects (*P* = n.s.).

Pre-operative, the presence of USWA was detected in 11 of 45 (24.4%). A comparison among healthy subjects and patients with CAPF showed a significant difference (*P* < 0.001).

At 12 month follow-up, the detected rate of USWA was lower in comparison to pre-operative values (*P* < 0.05) but they were not significantly higher as compared with healthy subjects. At 60 month follow-up the USWA values of CAPF patient were not statistically significantly different form the ones of healthy subjects.

### Complications and follow-up

There were no cases of urinary retention, anal stenosis or keyhole deformity. No necrosis of the transposed flap was observed. We did not report any local complications related to the injection of the BT. The only complications recorded post-operatively were of slight entity and in no case required further surgery; in particular, 3 infections were detected in the donor site and a partial break down of the flap occurred in one case.

## Discussion

The results of our study show that fissurectomy and anoplasty associated with the injection of BT, as treatment for patients affected by CAPF with IAS hypertonia, allows an immediate resolution of clinical symptoms as well as a fast healing of the wounds. We recorded a low rate of recurrence (6.7%); we observed only 3 “de novo” case of post-operatory anal incontinence, which were temporary and of slight entity; patients who were already suffering experienced a temporary worsening. Moreover, MRP values started significantly reducing from 12 months [10] after the procedure to reach, at 60 months after surgery, values which were similar to healthy subjects.

Fissurectomy is the most common surgical procedure used to preserve structural and functional integrity of the IAS. It consists in the in the excision of the CAF and it can be associated with the removal of the hypertrophied anal papilla and skin tags. Fissurectomy, as a wound debridement, removes the bradytrophic scar tissue and produces fresh wound edges, creating an acute fissure. Fissure excision without interfering with the IAS was first reported by Ashton in 1854, it was recognized to be an effective therapy for CAF, but it was abandoned due to its complications, such as keyhole deformity, which can lead to fecal incontinence [11]. Nevertheless, it has been recently reconsidered as a valid treatment for CAF both in children and adults [12]. This latter surgical procedure has been associated with pharmacological sphincterotomy to improve its results, as well as reduce its complications [15–20]. After surgical fissurectomy, with or without association with chemical sphincterotomy [13, 15, 21–26], we observe a complete second intention wound healing, even after 10 weeks and the rate of failure con reach the 34% [24]. The rate of recurrence reaches up to the 37% in some series [22]. The high rate of recurrence and healing failure of fissurectomy might be related to the fact that this surgical procedure leaves a naked ischemic area, whose previous blood supply arrives from some branches of rectal inferior arteries, which cross the hypertonic IAS fibers [27–29]; in this regard the employ of drugs enabling to reduce the IAS tone aims to improve the blood supply of the naked area.

The employ of a skin graft after fissurectomy was first described by Ruiz-Moreno in 1968.The use a flap to cover up for the naked area after fissurectomy is designed to relocate on this area healthy and fresh blood supplied tissue, perfused by other arterial districts. Another possible advantage of using a flap might be represented by the enlarging effect on the cutaneous circumference of the anal canal, which reduces the risk of splitting.

Several surgical techniques for the use of flaps have been described, we number among them the employ of skin or mucous flaps, skin ones are most frequently hired. Various type of skin flaps are known, such as sliding skin grafts, house advancement flap, V–Y advancement anoplasty, island advancement flap and rotation flaps [31–35].

Surgical procedures that involve the employment of flap guarantee shorter healing period and lower incidence of non- healed wound or recurrence and a minimal interference with the anal continence than the ones observed after surgical fissurectomy itself with or without association with pharmacological sphincterotomy [14, 30, 36, 37]. Fissurectomy associated with anoplasty is a surgical procedure employed for both patients with hypertonic IAS and normotonic IAS [35, 38]. Under the light of the brilliant results of this surgical procedure, some authors suggest using fissurectomy with anal advancement flap as the first line therapy for CAF [30, 39–41].

At the best of our knowledge, the association of fissurectomy with anoplasty and pharmacological sphincterotomy has been cited in one of our series [38, 42] and subsequently in a study published by Halahakoon et al. [43].

In this study, we associated the injection of BT in the IAS to the fissurectomy and anoplasty with V–Y skin advancement flap, to temporarily reduce the IAS pressure [44]. BT A prevents release of acetylcholine at the presynaptic nerve endings and blocks the neuromuscular transmission, thus causing a chemical enervation of the sphincter muscles. Nevertheless, this procedure is not free of risks, most frequently observed complications are peri-anal thrombosis, infections and anal incontinence. In this regard, we noticed that patients affected by pre-operative anal incontinence, experienced an immediate post-operatory worsening of the alteration, which diminished to pre-operatory level straight after 6 months; even for the 3 “de novo” cases, that we observed in the study, anal incontinence has been temporary and of slight entity but related to the BT action. The only surgical related complications we were able to record were of slight entity and they never required further surgery. Moreover, we recorded 3 cases of recurrence, which have not occurred at the same site as the original lesion and all healed with medical therapy; this might be because of the durability of the advancement flap [30].

However, during the fissurectomy with or without advancement flap, some steps may compromise the integrity of IAS. Therefore, it is mandatory to perform it with a scalpel to avoid excision or cauterization of the IAS fibers. It’s also useful not to use anal retractors, unless necessary and used for a brief period of time. In their prospective randomized clinical Zimmermann et al. [45] showed that trans anal advancement flap for high trans sphincteric fistula results in a significant reduction of MRP values and increase of anal incontinence rate after use of Park retractor when compared with the use of Scott retractor. Van Tets et al. [46] conducted a study in patients who underwent closed hemorrhoidectomy and showed that the reduction of MRP values was greater when a Park retractor was used; they also suggested that overstretching of anal sphincter by a Parks retractor may lead to small nerve branches rupture and consequently to the denervation of IAS fibers. As a matter of fact, in animal studies [47], it has been shown that prolonged stretching may cause local necrosis of external anal sphincter fibers. In view of these latter findings in our study we reached a good exposure of the anal canal just using 4 Kocher pliers placed at 3,6,9 and 12 h in gynecological position or just rarely using an anal retractor.

## Conclusion

At 5 years after the surgical procedure we achieved good results and observed that post-operative manometric findings were similar to those of healthy subjects; these evidences show us the surgical section of the IAS is not at all necessary for the healing process of the CAPF.

In conclusion, further randomized trials comparing fissurectomy alone versus fissurectomy associated with graft and drugs enabling the reduction of IAS tone are needed to better define their role in the treatment of CAPF with IAS hypertonia.

## Data Availability

The datasets generated during and/or analysed during the current study are available from the corresponding author on reasonable request.
